# Clinical Intervention Using Focused Ultrasound (FUS) Stimulation of the Brain in Diverse Neurological Disorders

**DOI:** 10.3389/fneur.2022.880814

**Published:** 2022-05-09

**Authors:** Hongchae Baek, Daniel Lockwood, Emily Jo Mason, Emmanuel Obusez, Matthew Poturalski, Richard Rammo, Sean J. Nagel, Stephen E. Jones

**Affiliations:** ^1^Cleveland Clinic, Imaging Institute, Cleveland, OH, United States; ^2^Center for Neurological Restoration, Cleveland Clinic, Neurological Institute, Cleveland, OH, United States; ^3^InSightec, Inc., Tirat Carmel, Israel

**Keywords:** clinical focused ultrasound, MRgFUS, high-intensity focused ultrasound (HIFU), low-intensity focused ultrasound (LIFU), thermoablation, neuromodulation, blood-brain barrier (BBB) opening

## Abstract

Various surgical techniques and pharmaceutical treatments have been developed to improve the current technologies of treating brain diseases. Focused ultrasound (FUS) is a new brain stimulation modality that can exert a therapeutic effect on diseased brain cells, with this effect ranging from permanent ablation of the pathological neural circuit to transient excitatory/inhibitory modulation of the neural activity depending on the acoustic energy of choice. With the development of intraoperative imaging technology, FUS has become a clinically available noninvasive neurosurgical option with visual feedback. Over the past 10 years, FUS has shown enormous potential. It can deliver acoustic energy through the physical barrier of the brain and eliminate abnormal brain cells to treat patients with Parkinson's disease and essential tremor. In addition, FUS can help introduce potentially beneficial therapeutics at the exact brain region where they need to be, bypassing the brain's function barrier, which can be applied for a wide range of central nervous system disorders. In this review, we introduce the current FDA-approved clinical applications of FUS, ranging from thermal ablation to blood barrier opening, as well as the emerging applications of FUS in the context of pain control, epilepsy, and neuromodulation. We also discuss the expansion of future applications and challenges. Broadening FUS technologies requires a deep understanding of the effect of ultrasound when targeting various brain structures in diverse disease conditions in the context of skull interface, anatomical structure inside the brain, and pathology.

## Introduction

Focused ultrasound (FUS) is a transformative tool that can be used to noninvasively create lesions or temporarily modify the function of targeted brain tissue while minimally affecting all intervening tissues carrying the ultrasound energy. Because FUS can be used to create these lesions remotely from the source, with well-defined margins and precise localization, this technology is an attractive option for noninvasive neurosurgery (1).

The field of FUS was created in 1927 by Wood and Loomis, who first documented the effects of ultrasound on living biological tissue (2). Therapeutic applications for high-intensity focused ultrasound (HIFU) often use a lower frequency (300 kHz – few MHz) with maximum ultrasound intensity at a beam focus of approximately 1,500 W/cm^2^, whereas a high frequency range (2–15 MHz) and low intensity (0.1 W/cm^2^) are typical for diagnostic ultrasound (3). A drawback of the early animal experiments in the field of HIFU was the considerable tissue damage caused along the ultrasound pathway from the skin to the target. This damage was partly due to the use of a single source, which provided no geometric gain when compared with current technology. A further complication in the creation of brain lesions was the presence of the skull, which is a source of sound wave reflection, scatter, and absorption; all of these factors reduce power deposition at the target. Although the source power could be sufficiently increased to overcome this loss and create deep brain lesions, the increased power would also increase collateral damage to the scalp and skull. Thus, early animal work necessitated removal of skull flaps, thereby limiting clinical application of this technology.

**Figure 1:**
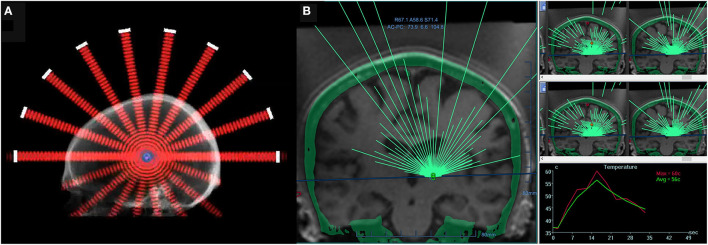
**(A)** A 2-dimensional schematic of a hemispherical phased-array transducer, showing multiple beams converging at a geometric focus. **(B)** A screen image of a commercial software MRgFUS with >1,000 individual transducer elements (ExAblate; InSightec, Israel). All transducer elements use a phase shift algorithm to account for individual skull effects. Transducer elements are individually electronically steered to ensure precise, submillimeter targeting. For the treatment of essential tremor, a typical device operates at approximately 650 kHz, achieving a focal spot size of approximately 6 mm and a target temperature of 55°C (right bottom panel: real-time temperature monitoring via MR thermometry), thus allowing for thermoablation of a deep-seated brain structure.

Considerable progress in human brain HIFU was made in the 1950s by the Fry brothers, who developed a 4-beam technology and demonstrated the therapeutic potential of FUS for treating neurological disorders by creating lesions deep inside a primate brain (4). Although these advances demonstrated the great potential of HIFU for treating diverse neurological diseases, successful clinical application would require real-time imaging to accurately visualize and verify target location. To this end, a multi-element phased-array system (Figure 1) was combined with magnetic resonance imaging (MRI), thus allowing for MR-guided FUS (MRgFUS) (5–7). Starting with *in vitro* studies in 1998, this technique permitted simultaneous visualization of anatomical and temperature maps and provided the feedback needed to perform a completely incisionless and closed-loop procedure.

**Figure 2:**
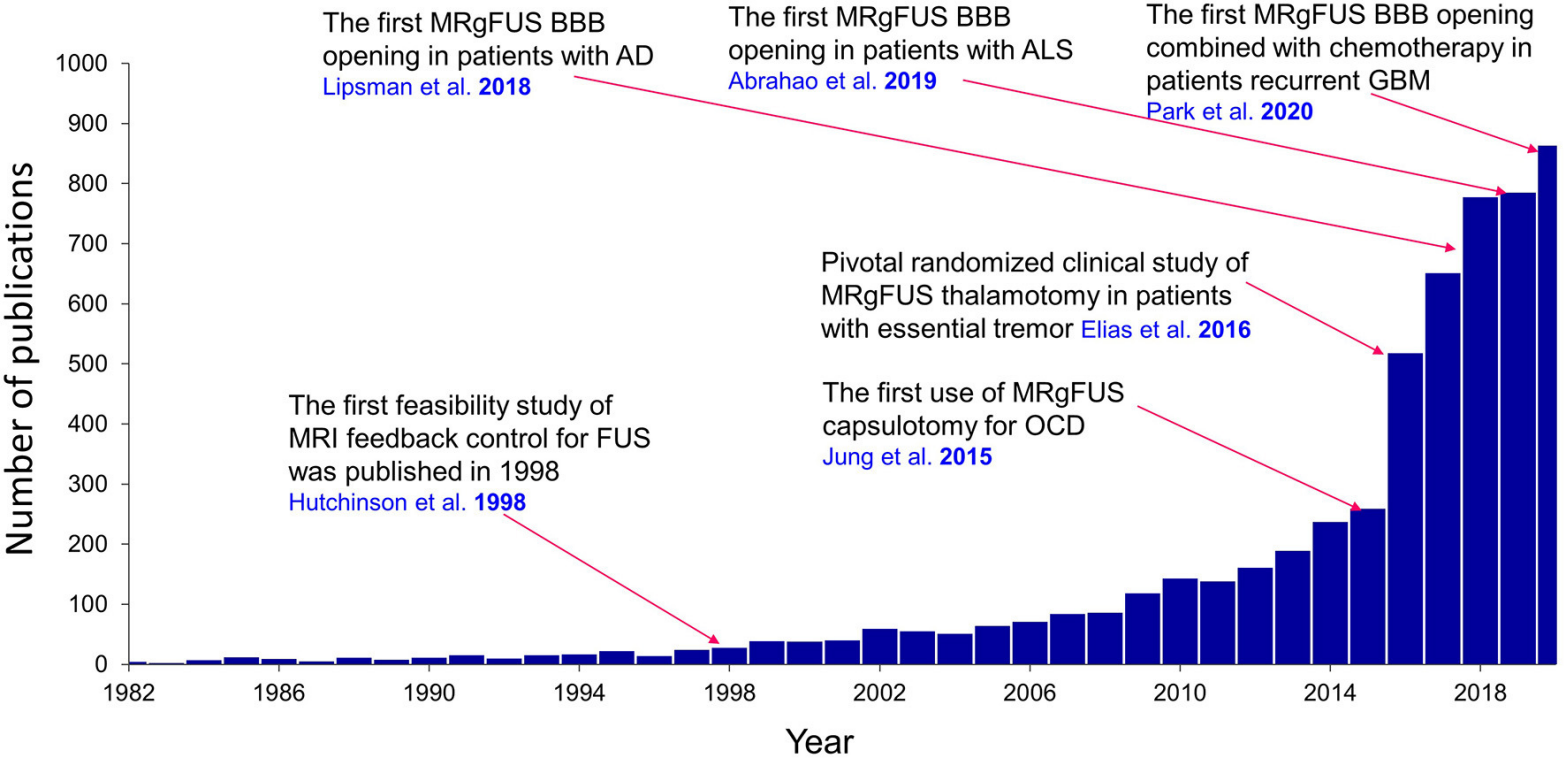
Publications regarding the clinical application of FUS in neurological disorders. A landmark study for each clinical application is overlaid on the graph. Results were obtained from PubMed. AD, Alzheimer's disease; ALS, amyotrophic lateral sclerosis; BBB, blood-brain barrier; GBM, glioblastoma multiforme; OCD, obsessive-compulsive disorder.

In 1998, Hynynen and Jolesz (8) reported using pretreatment computed tomography (CT) scans to inform a phased-array HIFU system with phase-correction methods to further mitigate skull attenuation by tightening the focus, thereby increasing the energy deposition density. This approach led to Food and Drug Administration (FDA)–approved MRgFUS system that uses thermoablation to treat essential tremor (ET) and tremor-dominant Parkinson's disease (PD) in 2016, and more recently FDA approved thermoablation of internal globus pallidus, pallidotomy, as an alternative MRgFUS treatment for PD dyskinesia in November 2021. Research to expand the clinical application of FUS technology to other neurological disorders has since increased greatly (Figure 2).

Because of the diverse biophysical properties of ultrasound, the effects of FUS on biological tissue may include heat, cavitation (both stable and unstable), histotripsy, microbubble interactions, and both low-intensity and high-intensity microstreaming. Various therapeutic FUS applications can exploit these bioeffects, allowing clinicians to perform thermoablation, immunotherapy, histotripsy, opening of the blood-brain barrier (BBB), and neuromodulation. Many *in vitro* and *in vivo* studies have evaluated the feasibility and safety of these applications for a variety of diverse neurological conditions (Figure 2). In this review, we will discuss the well-established clinical use of MRgFUS for the treatment of ET, PD, and obsessive-compulsive disorder (OCD), as well as current clinical trials assessing the use of FUS methods for the treatment of glioblastoma, Alzheimer's disease (AD), amyotrophic lateral sclerosis (ALS), and epilepsy.

## Fus-Mediated Thermal Ablation

### Effect of the Physical Properties of the Skull on MRgFUS Thermal Ablation

If a high enough temperature can be reached to create a thermoablative lesion of the correct size and at the correct location, considerable clinical efficacy can be obtained for conditions such as PD (9), ET (10), OCD/major depressive disorder (MDD) (11), and epilepsy (12). Although modern MRgFUS techniques generally allow high spatial and temporal resolution of temperature characterization and effective control of temperature distribution in the brain, several studies have reported difficulties in achieving a temperature that reaches the ablative level in patients with ET and PD because of the physical properties of the skull (13, 14).

The skull's acoustic properties are different from those of soft tissue. The intrinsic attenuation of ultrasonic waves in the skull is ~20 dB/cm^*^MHz, which is higher than the attenuation of waves in brain tissue (~0.8 dB/cm^*^MHz) (15). Attenuation arises from acoustic absorption and scattering in all directions in the medium. Acoustic scattering refers to that part of an incident acoustic wave that is reflected from interfaces between different tissues due to inhomogeneities in their density and compressibility (16, 17). This scattering can be substantial at major interfaces, such as between bone and soft tissue. Reflection is also high at interfaces between the outer/inner tables of cortical bones and the central cancellous bone because of their different bone structures (18). Increased attenuation implies decreased heating power available at the target, in addition to increased deposition at nontarget soft tissues such as the scalp and skull. Such unwanted heating is exacerbated if the total incident power is increased to compensate for increased attenuation and maintain the desired temperature at the target. There can be great variability in attenuation between portions of a single skull and also between the skulls of different patients. Some patients are effectively untreatable with this method because of potential scalp burns or damage to the underlying bone, in addition to the painful heating of nontarget tissues. Thus, a practical simple measure that can predict which patients may benefit from HIFU is needed.

**Figure 3:**
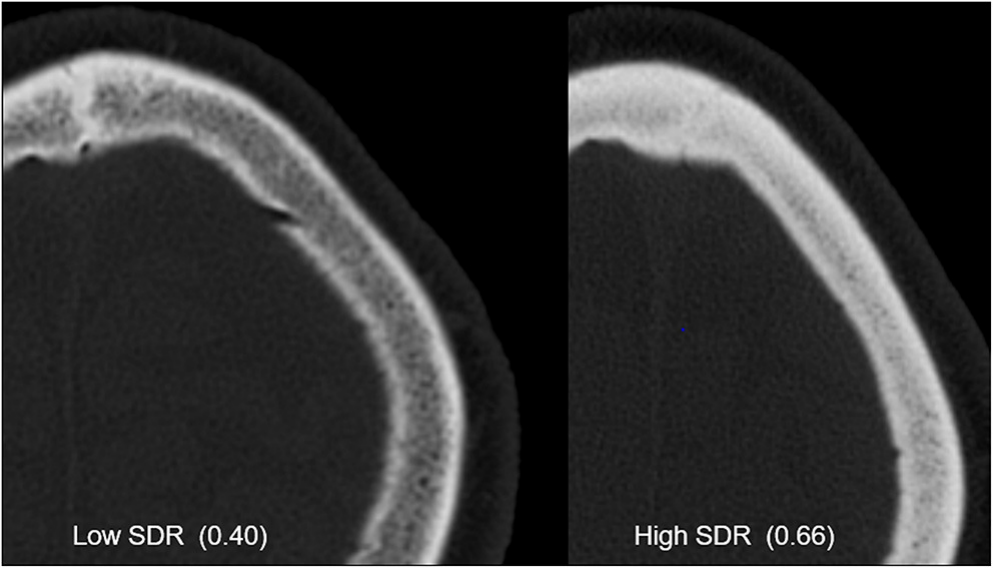
CT images from patients with low and high skull density ratios (SDRs). Both images are windowed the same way, and the skull with high SDR clearly shows increased Hounsfield units.

Along any ultrasound ray traversing the skull, one can calculate the ratio of skull density between the mean cortical and mean cancellous bone using the Hounsfield units (HUs) that result from a CT scan with high resolution and using a bone kernel for image reconstruction. These ratios can be averaged over all rays traversing the skull in a HIFU configuration to provide a single measure called the skull density ratio (SDR), which is a useful global index for identifying patients who are eligible to undergo MRgFUS-mediated lesion creation (Figure 3). Although currently used MRgFUS techniques can compensate for skull factors with CT-based phase-correction software on multiarray systems, some patients will still demonstrate sufficiently low SDR to restrict them from treatment. To this end, practice guidelines from the American Society for Stereotactic and Functional Neurosurgery state that patients with an SDR <0.4 should not undergo MRgFUS for lesion creation (19), as insufficient heating at the intracranial target will lengthen the time needed to achieve ablative temperatures and lead to excessive heating at nontarget tissues.

Researchers have also assessed other factors such as skull morphology (20) and volume (21) as potential predictors of MRgFUS treatment success. For instance, in a retrospective analysis of 189 patients who underwent MRgFUS, D'Souza et al. (22) found that patients across different SDR categories (SDR <0.4, 0.4 ≤ SDR <0.45, and SDR ≥ 0.45) demonstrated similar improvements in clinical outcomes indexed by 1-year follow-up Clinical Rating Scale for Tremor (CRST) scores even though the percentage of patients achieving the peak temperature of 54°C was substantially higher in patients with SDR ≥ 0.45 (91%) than in those with 0.4 ≤ SDR <0.45 (64%) and those with SDR <0.4 (55%). Several other studies have reported cases of patients with low SDR in whom permanent ablation was still achieved (23, 24). Thus, there is an urgent clinical need to investigate factors such as skull thickness, incidence angle, and skull heterogeneity as potential predictors of treatment success, thereby identifying new metrics that may be more accurate in predicting success among patients with low SDR.

**Figure 4:**
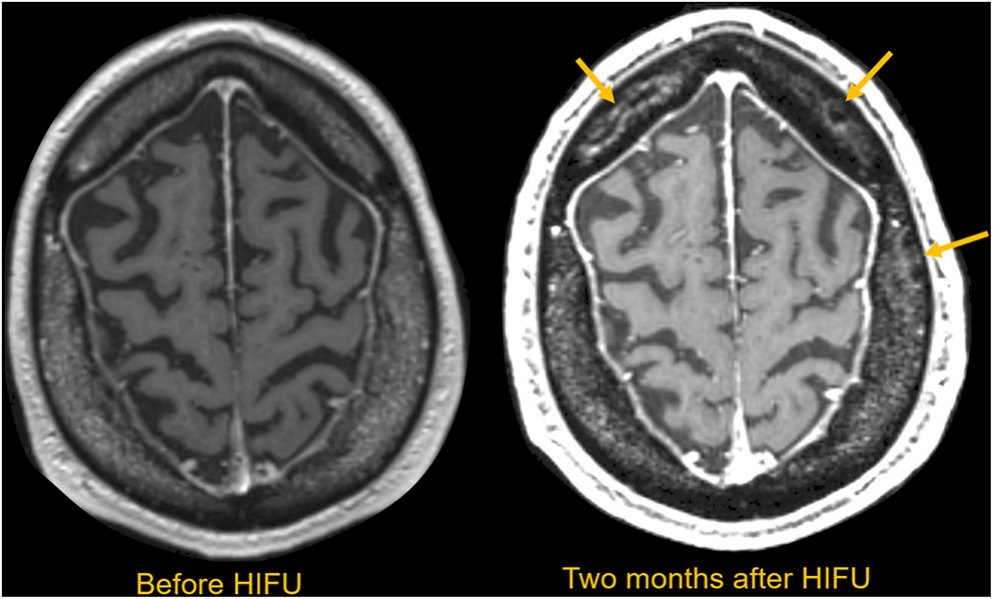
MRI scan obtained before and 2 months after HIFU in a patient requiring extended sonications to make a durable thalamic lesion. Multiple serpiginous marrow lesions compatible with infarctions are subsequently seen in the marrow space, as indicated with yellow arrows. These lesions are asymptomatic, but notable.

For patients with low SDR, the thalamic target energy deposition will be inefficient, leading to an increased risk of overheating the scalp and skull at the expense of therapeutic heating in the thalamic target. This unwanted heating will cause the patient pain and could lead to irreversible tissue damage (Figure 4). In addition, the efficiency of energy deposition decreases over the course of multiple treatment cycles, potentially due to effects from skull heating (25). A recent study of patients treated with MRgFUS reported that seven out of 30 patients demonstrated multiple new asymptomatic calvarial marrow injuries 3 months after attempted treatment (26). This study found no correlation between SDR and the presence of skull lesions, but the maximum power used was substantially higher for patients with lesions than for those without. For instance, one patient with a skull lesion had undergone prolonged sonication for 31 s with 1,100 W maximum power, but the maximum temperature achieved was only 48°C. There is a well-established time-temperature relationship that can change various time-temperature profiles into a standardized single measurement to estimate the degree of the thermal dose while allowing for tissue necrosis (27). For future MRgFUS procedures, clinicians must be able to tailor parameters such as maximum energy, sonication duration, and number of sonication sessions so that they can prevent thermal hotspots in the skull and thus prevent long-term skull injuries, especially for patients with unfavorable skull bone characteristics.

InSightec's hemispheric phased array system is composed of 1,024 element of transducers, and besides the skull bone property, i.e., SDR and thickness, the successful delivery of ultrasound beam from each transducer depend on the angle of incidence to the skull surface. Since the shape of the skull is not spherical, if the targeting brain structure is located far from the center of the brain, some of the ultrasound rays may likely have an incidence angle to the skull of >25°. The increased angle of incidence will deactivate the corresponding transducer and lead to a less cumulative number of active elements as the incidence angle of >25° will increase reflection and thereby likely decrease energy deposition inside of the planned target and increase deposition in the scalp. As a specific example, a recent study by Jung et al. (28) showed that an increased number of elements were deactivated when targeting globus pallidus interna (Gpi) and anterior limb of the internal capsule (ALIC), which are more lateral brain structures from the center of the brain compared to the thalamus. Several simulation studies have suggested that significant attenuation may be attributed from longitudinal-shear (transverse) mode conversion (29, 30); however, another study also reported that the conversion of longitudinal waves to shear waves inside the skull is insignificant when the incidence angle is <20°, with the assumption that amplitude loss during shear wave conversion from incident rays at the skull is not critical (31). An investigation using an *ex vivo* human skull demonstrated a significant reduction (nearly 31% loss of normal incidence) in transmitted amplitude when the incident angle was 31° at 0.548 MHz (32). In the study by Jung et al. (28), at an incidence angle >25°, energy transmission sharply decreased when the SDR was <0.6, but the energy transmission started to recover when the SDR was >0.6, indicating that a high SDR compensates for the influence of a higher incidence angle. This study highlights that even though SDR provides a useful standard value for screening eligible patients, the role of incidence angle also must be considered, especially when the focal region is distant from the transducer's geometric focus as in cases of capsulotomy (33) and pallidotomy (34).

Jung at al. (28) also demonstrated higher energy transmission (by a factor of ~3) at a lower frequency (230 kHz) than at a mid-frequency (680 kHz) for all SDR and incidence angle ranges. These findings helped to initiate subsequent studies developing a low-frequency system to circumvent skull limitations at higher frequencies, in addition to broadening the regions in the brain accessible to lesion creation.

Recent studies have used computer simulations to better predict the temperature increase in targets in individual skulls by modeling the skull efficiency with properties extracted from CT, in particular the HU (36, 37), an arbitrary unit of radio density. Although the HU has widespread applicability, it is still dependent on various other factors (38), and so standardization of CT parameters should optimize the use of HU as a rigorous diagnostic tool for evaluating skull adequacy for MRgFUS lesioning. Recently, researchers reported the use of a novel method employing microbubbles as an ultrasound contrast agent; this technique allowed acoustic echoes to modify phase corrections and thereby narrow the acoustic focus. This method, called “echo focusing,” provided sonication efficiency for lesion formation that was superior to that obtained with CT-based aberration correction (24, 39). In these studies, an echo-focusing phase aberration correction technique was incorporated by measuring returning acoustic signals from intravenously injected microbubbles around the intended target region during sonication (40, 41). With echo focusing, successful lesion formation was achieved in 12 patients with ET, including 3 patients in whom MRgFUS thalamotomy treatment using CT-based aberration correction had failed (24). In another study, 8 patients with low SDR (mean SDR = 0.35) were successfully treated using the echo-focusing method by raising the temperature to >54°C in patients with ET and to >52°C in patients with PD; these temperatures were sufficient for lesion formation (39). This echo-focusing technique could be particularly beneficial for patients with low SDR and for those with a target that is more lateral than the thalamus, as this research demonstrated permanent lesion formation in cases of pallidotomy in patients with an SDR <0.4.

### Intraoperative MRI and Accelerometer Measurements to Guide Treatment

**Figure 5:**
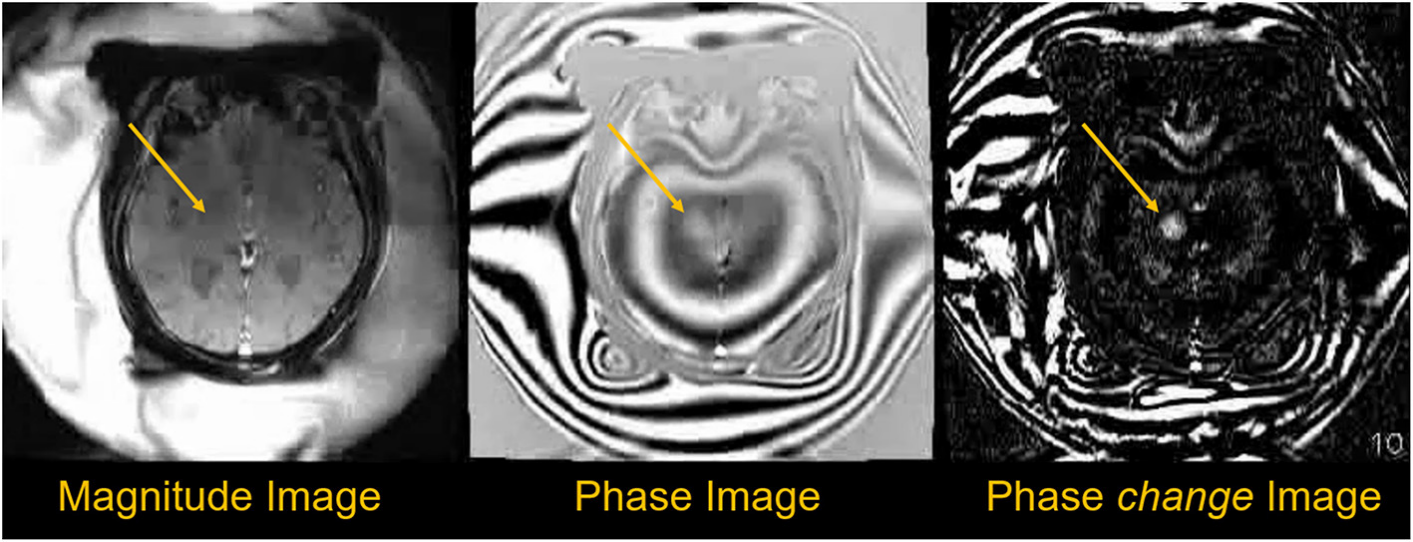
Phase data used to obtain temperature information during sonication. The magnitude image shows little change during treatment, but the phase images show more measurable changes. Temperature difference maps (ΔT) can be created using phase-change images obtained every ~6 s during treatment, with temperature changes proportional to cumulative phase shift (35).

**Figure 6:**
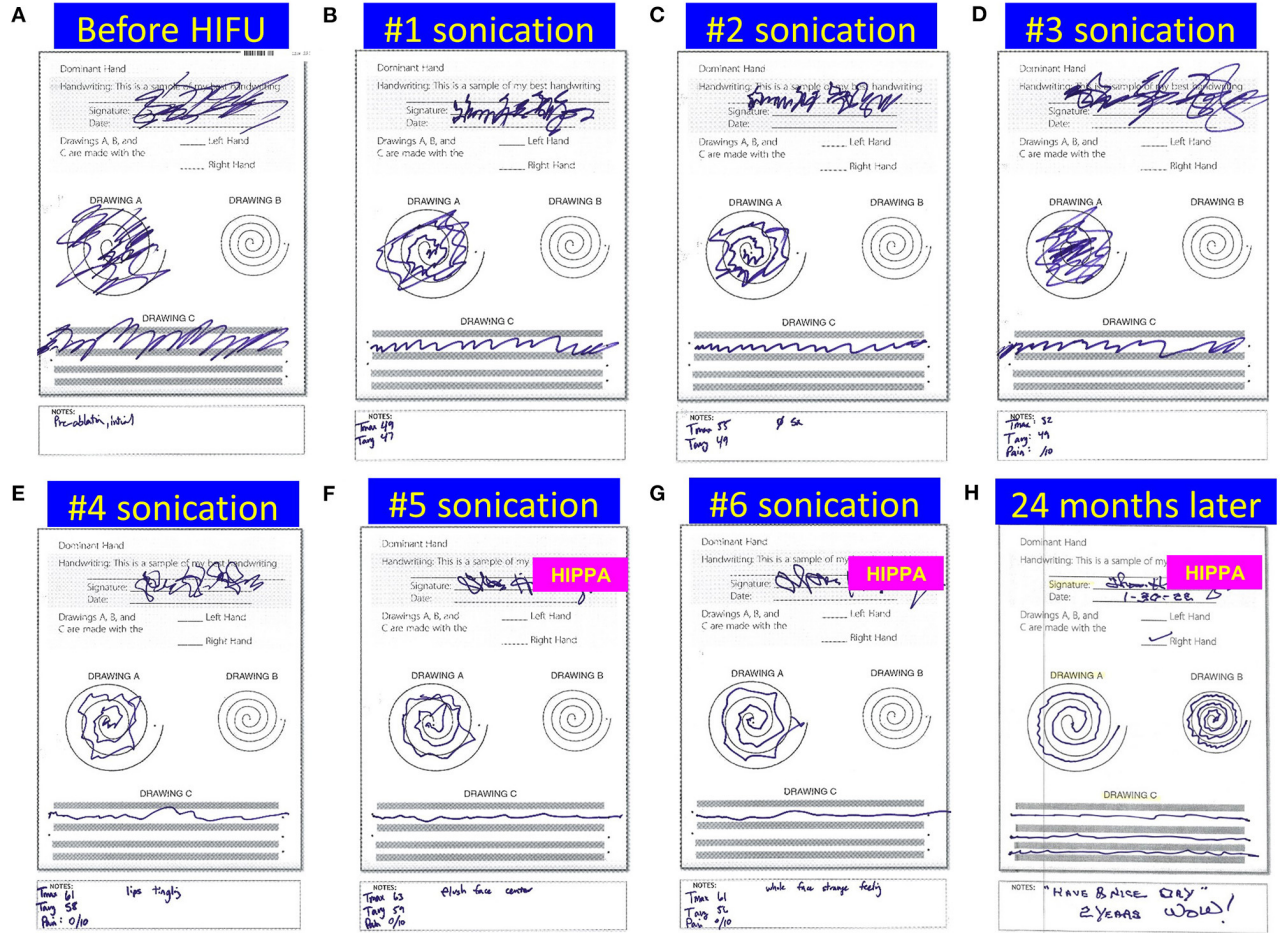
Drawings from a patient with essential tremor during MRgFUS left thalamotomy. The patient's handwriting, spiral, and line drawing were checked after each sonication in their supine position inside the MR suite **(A–G)**, and we received 2-year follow-up mail of handwriting sheet from the patient **(H)**. Note the handwriting of the patient's name after HIFU has been blocked for deidentification purposes because it is now sharp enough to read.

**Figure 7:**
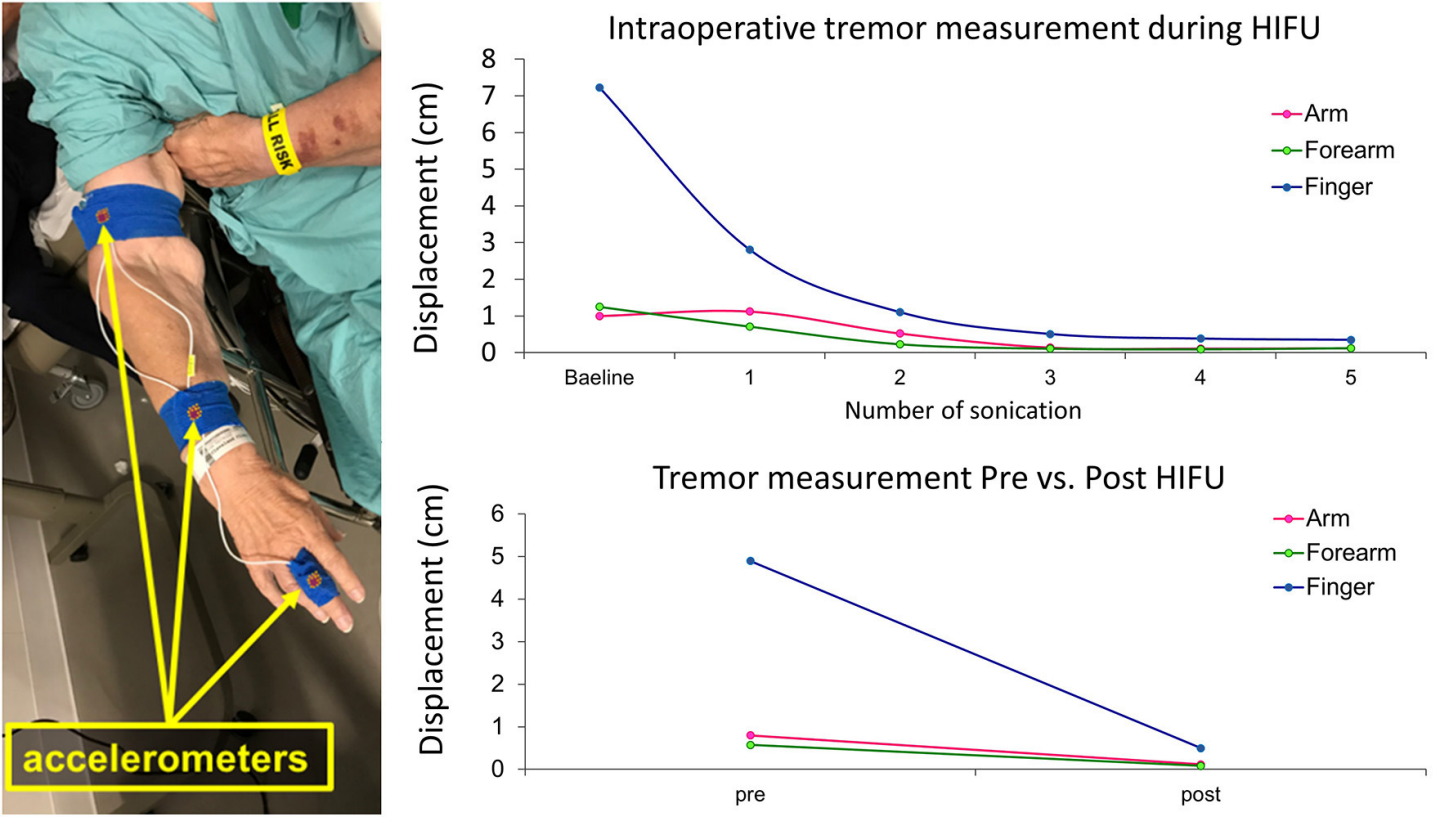
Intraoperative monitoring of treatment response using accelerometer recordings. Accelerometer sensors are placed on the patient's finger, forearm, and upper arm during MRgFUS thalamotomy. With the patient in the MR machine, displacements of the finger, forearm, and upper arm during tremor are plotted after each sonication measure (upper graph). With the patient sitting upright in the preparation room, postural tremor is measured before and after the thalamotomy procedure (lower graph).

Similar to deep brain stimulation (DBS), FUS has features that confer “closed-loop” status. Specifically, during the staged procedure, repeated examinations of the effect of increasing sublesional temperatures on the patient's tremor (as measured with continuous MR thermometry; Figure 5) (35) provide near–real-time feedback to verify targeting, monitor outcome, and update the treatment plan. Additionally, because the patient does not need to be placed under general anesthesia for FUS, the effect of treatment on tremor can be observed immediately after each sonication both from the accelerometer and the patient's handwriting (Figure 6). Approaches such as the use of an intraoperative accelerometer to quantify the tremor response in real time are necessary and will help to complete closed-loop feedback procedures in a patient-specific manner (Figure 7).

**Figure 8:**
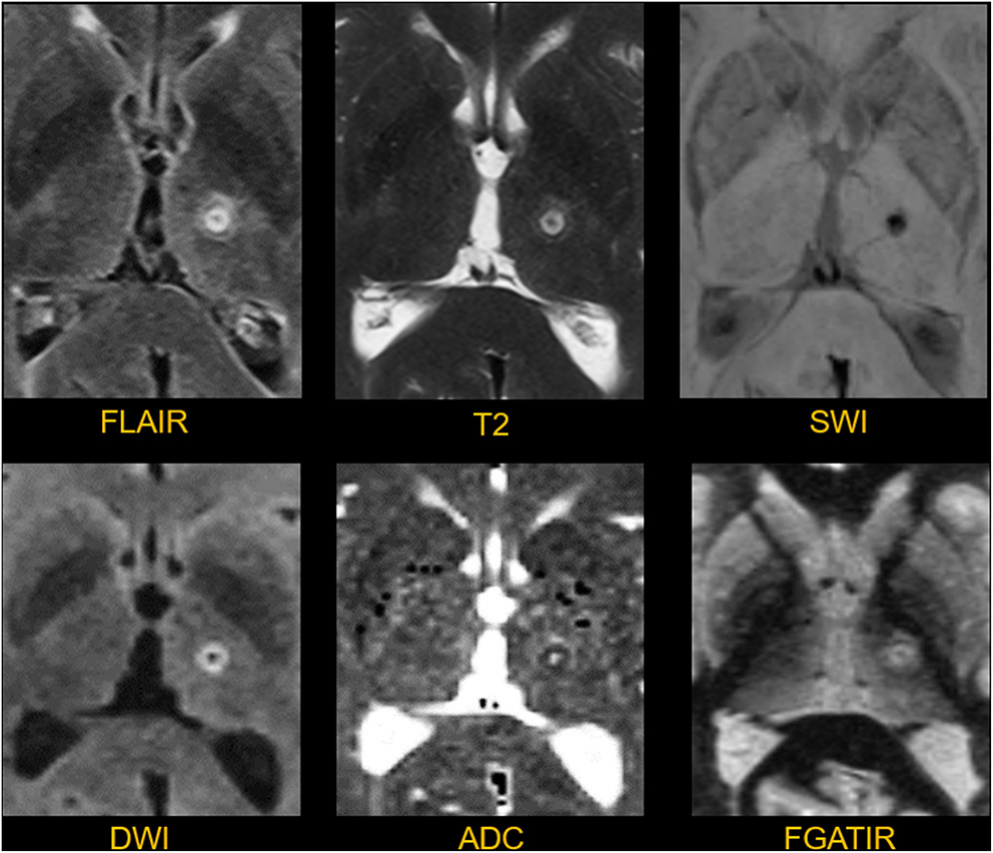
Appearance of a thalamic lesion on postoperative day 1. The lesion in the left thalamus is manifest as a T2/FLAIR spherical hyperintense focus with a diameter of about 6 mm. There is hemosiderin clearly shown in the susceptibility weighted image (SWI). There is a rim of restricted diffusion with a core of facilitated diffusion shown on the DWI and ADC images. The FGATIR sequence shows the relationship between the lesion, thalamus, and adjacent internal capsule.

**Figure 9:**
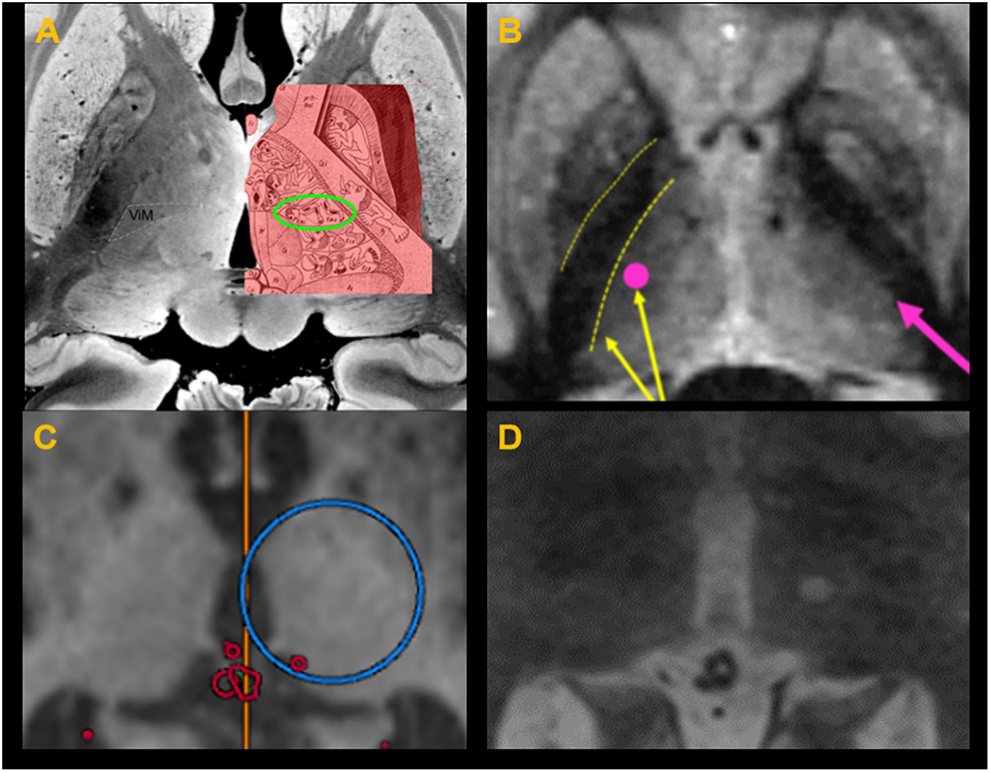
**(A)** Thalamic homunculus superimposed over the thalamus in post mortem ultra–high-resolution images obtained at 7T. The green circle indicates the location of the ventral intermediate nucleus (Vim). **(B)** Fast gray matter acquisition T1 inversion recovery scan shows sharp demarcation between the IC (internal capsule) and thalamus, allowing adjustment of the target selection to avoid lesion spread into the IC. **(C)** Target coordinate selection during treatment planning using the anterior commissure–posterior commissure as standard references for localizing the target. **(D)** A successful lesion is created at the Vim, confirmed with a T2-weighted image obtained immediately after sonication with the ablative temperature.

**Figure 10:**
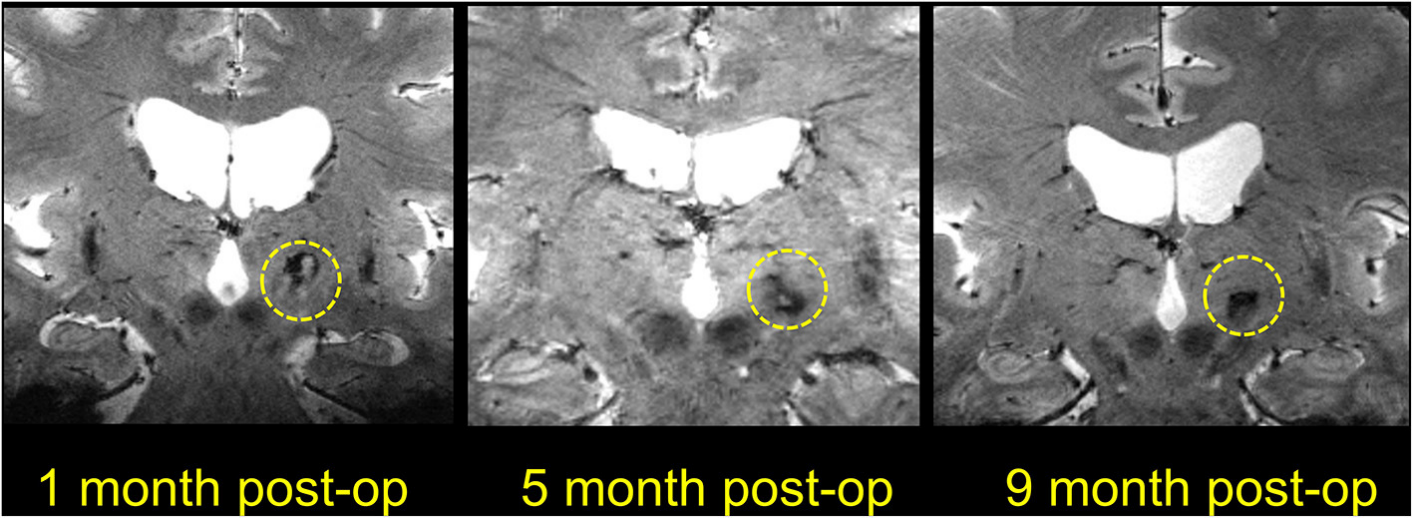
Coronal T1-weighted 7T images showing lesions at different time points after MRgFUS thalamotomy. The three images are all from different patients, as the postoperative MR images were obtained at different time points.

Research should continue to focus on developing a reliable method to identify the target, reduce lasting side effects, and enhance durability. For instance, during the course of the MRgFUS thalamotomy, different MR sequences can provide information about lesion volume and diameter changes over time (Figure 8). Only T2-weighted sequences can show the lesion shortly after it is created; however, the lesion may not be apparent on T2-weighted images obtained up to 180 days after the procedure. Susceptibility-weighted images, on the other hand, can demonstrate the lesion up to 180 days after treatment (42). Fast gray matter acquisition T1 inversion recovery imaging can be used for surgical planning, as this method offers superior visualization of the target and is especially effective in differentiating between the internal capsule and thalamus (Figure 9). Furthermore, T1-weighted 7T images can depict lesion shrinkage and shifting up to 65 days after treatment (Figure 10).

### Movement Disorders

Disabling movement disorders such as ET and PD are often diagnosed in patients of advanced age, and the incidence of these disorders is increasing due to a growing and aging world population. The effect of movement disorders on daily life is considerable, impairing routine functions such as holding a glass of water, writing a check, or using a hand-held device. Safe and effective treatment options are therefore needed so that patients can maintain independent function. Although medications such as beta-blockers may adequately control mild upper extremity tremor, they are practically inconsequential for slowing moderate to severe tremor. Propranolol and primidone (beta-Blockers) are the most common choice of drugs in medical therapy for treating moderate to severe functional disability in ET. However, if the patient has a contraindications to beta-blockers or inadequate tremor control, other drugs such as Mysoline, Benzodiazepines, gabapentin, topiramate, zonisamide can be used as add-on therapy or monotherapy. Surgeries such as FUS-mediated Vim thalamotomy or DBS are considered last when medical treatment does not help suppress tremor. On the other hand, PD has more complex features encompassing both motor and non-motor disabilities. Most PD patients initiate treatment with levodopa therapy, the most effective drug in treating PD. However, the long-term use of levodopa frequently leads to dyskinesia (43) and wearing-off phenomenon. Patients with levodopa resistance or with rapid progression motor symptoms seek surgical treatment such as bilateral DBS, or unilateral FUS-mediated Vim thalamotomy. They could also benefit from recently approved FUS-mediated pallidotomy for bradykinesia and drug-induced dyskinesia, etc.

DBS of targets such as the ventral intermediate nucleus (Vim) of the thalamus (44), subthalamic nucleus (45), and internal globus pallidus (46) is a well-established option for the treatment of movement disorders. The benefits of this treatment are long-lasting and the risk profile is low; however, the procedure is costly and invasive and requires permanently implanted hardware. Because FUS circumvents these surgical complications, there was early enthusiasm that this technique might appeal to patients reluctant to undergo DBS. Radiosurgery using Gamma Knife had a similar appeal, but this procedure was limited by an absence of intraoperative validation (47) and a lack of reliable methods for targeting.

The overall outcomes of FUS and radiosurgical thalamotomy for tremor are related to the size and location of the lesion; these factors also govern potential side effects. While the durability and efficacy of treatment may be greater with larger lesions, so too is the probability of adverse effects (10). Radiosurgical thalamotomy has a shallower temperature gradient than HIFU (48), which consequently increases the uncertainty of the lesion's margins and increases the risk of extending the lesion beyond the planned target. Although the temperature range with HIFU may be easier to control, there is a small lag (2–3 s) in MRI thermometry maps, which may partially offset this advantage.

In 2016, based on data from a clinical trial by Elias et al. (14), the FDA approved the use of HIFU for ablation of the Vim in patients with ET, and this indication was expanded in 2018 to include ablation of the Vim in patients with tremor-dominant PD. With this technique, high-intensity ultrasound waves irreversibly create lesions in the target structures *via* coagulative necrosis in the tissue secondary to the heat resulting from frictional forces (49, 50). The temperatures typically need to exceed 55°C for this treatment to be effective (51). In the first proof-of-concept clinical study testing HIFU in a randomized controlled trial, ablation of the thalamic Vim with MRgFUS significantly suppressed tremor, with patients demonstrating improvements in finger-to-nose pointing tasks (14). In addition, scores on the CRST were reduced by 81.3% at 3 months after HIFU treatment compared with baseline scores. A follow-up study published in 2018 (52) demonstrated a sustained effect at 2 years in most patients with ET, and another follow-up study highlighted continued benefit from the unilateral thalamotomy after 3 years (53). It is also noteworthy that patients who benefited from the unilateral MRgFUS thalamotomy without experiencing any side effects wished to extend the lesioning procedure for bilateral thalamotomy to improve on the other side. Although a few case reports of bilateral thalamotomy state that staged second treatment were successful without severe adverse events (54–57), bilateral thalamotomy is not currently approved as standard treatment as the research of bilateral thalamotomy in relation to the tremor etiology, adjustment of the second lesion, and overall incidence of adverse events are still under investigation (NCT04112381, https://clinicaltrials.gov/ct2/show/NCT04112381).

Since this initial research was published, multiple other studies have demonstrated similar findings. For instance, Bond et al. (9) used MRgFUS to perform unilateral Vim thalamotomy in patients with PD refractory to levodopa and reported a 62% improvement in CRST scores for hand tremor contralateral to the treatment side. In another study, MRgFUS of the Vim led to a 55.9% improvement in tremor score at 3 months after treatment in 6 patients with diverse tremor types including PD, dystonia, dystonia gene–associated tremor, and writer's cramp (58). Two patients experienced lasting side effects of hemitongue numbness and hemiparesis with hemihypoesthesia, suggesting the need for long-term safety evaluation with a larger sample size. In 2020, researchers assessed the feasibility of using MRgFUS for unilateral pallidotomy in PD patients with dyskinesia induced by the long-term use of levodopa (34). The study demonstrated a 43% improvement from baseline in Unified Dyskinesia Rating Scale score, an effect that persisted through 12 months. As the pathophysiology of PD is different from that of ET, patients show distinct cardinal signs such as rest tremor, rigidity, and bradykinesia, which has prompted researchers to explore novel therapeutic targets for PD. In November 2021, FDA extended the therapeutic target for PD and approved MRgFUS for pallidotomy. Additionally, a clinical trial assessing bilateral ablation of the pallidothalamic tract for PD is currently ongoing (NCT04728295, https://clinicaltrials.gov/ct2/show/NCT04728295). Furthermore, researchers in Madrid assessed lesioning the subthalamic nucleus for PD with markedly asymmetric signs and found that the Movement Disorder Society-Unified Parkinson's Disease Rating Scale motor score (i.e., part III) was decreased by approximately 50% from baseline to 4 months after treatment (59).

Although early research studied the response of tremor to HIFU targeting of the Vim, other targets are also under investigation for the treatment of disabling movement disorders. For example, the internal globus pallidus is a preferred target when patients' symptoms are dominated by dyskinesia and dystonia. Previous research has demonstrated that pallidotomy and pallidal DBS lead to marked improvements in the symptoms and motor dysfunctions of PD (60).

The current evidence suggests that HIFU is a safe and effective option for patients with disabling ET or PD who are not candidates for DBS or are reluctant to undergo surgery. However, more studies are needed to address the nontremor motor symptoms of movement disorders. Additionally, questions regarding durability, the safety of bilateral treatment, and novel therapeutic targets for tremor are currently being investigated in clinical trials.

### OCD

OCD is a common psychiatric disorder characterized by repetitive behaviors, compulsions, and urges detrimental to health and quality of life (61). Selective serotonin reuptake inhibitors are currently the first-line pharmacotherapy for management of OCD. Because of the chronic nature of this disease, medical therapy is often combined with cognitive and behavior therapy to increase the durability of treatment. However, 20% to 30% of patients do not respond to medication and could potentially benefit from neurosurgical options such as DBS (62, 63), radiosurgery (Gamma Knife) (64), and MRgFUS (33). Of these techniques, MRgFUS has the advantage of being noninvasive, with the added benefit of lack of general anesthesia and associated surgical complications. In addition, with MRgFUS, the lesion size and location can be controlled in real time.

The study of MRgFUS in psychiatric disorders dates back to the 1950s, when neurosurgeon Petter Aron Lindstrom first removed brain tissue using MRgFUS as an alternative to prefrontal invasive craniotomy and lobotomy (65). Lindstrom introduced the concept of MRgFUS-mediated lobotomy to his colleague Lars Leksell, who then set out to study the use of MRgFUS for treating psychiatric disorders (Steiner L. Personal communication. 2007). Leksell designed a custom stereotactic headframe as a precise lesioning tool but was unable to complete his MRgFUS investigations because of the challenges imposed by the transmission of ultrasound through the skull. However, his contributions to developing a noninvasive modality for the treatment of functional brain disorders laid the foundation for the development of the first Gamma Knife model.

Research into the pathological brain networks responsible for OCD has focused on the cortico-striatal-thalamo-cortical pathway (33), and recent neuroimaging studies suggest involvement of the orbitofrontal cortex, the dorsal anterior cingulate cortex, and the amygdalo-cortical circuit (66, 67). In a study of 4 patients with OCD, bilateral MRgFUS was used to create lesions in the anterior limb of the internal capsule. This dense white matter tract consists of afferent and efferent fibers of the affect network and reward network that run their course up to the orbitofrontal cortex (68) and form a target for medication-resistant OCD. The patients treated with this procedure demonstrated a gradual improvement in the Yale-Brown Obsessive-Compulsive Scale (Y-BOCS), with a mean improvement of 33% over a 6-month follow-up (33). In addition, depression and anxiety levels after MRgFUS capsulotomy were almost immediately improved (mean reductions of 61.1 and 69.4%, respectively). Similarly, Kim et al. (69) used MRgFUS bilateral capsulotomy to treat 7 patients with medication-refractory OCD and measured the patients' Y-BOCS scores at 1 week and at 1, 3, 6, 12, and 24 months. Significant improvement (38%) from baseline was seen at the 24-month follow-up, without any severe adverse events. In addition, OCD symptoms started to improve as early as 1 week after MRgFUS capsulotomy, and depression and anxiety levels were also reduced at 1-week follow-up (−47.4% on the Hamilton Rating Scale for Depression and −53.6 % on the Hamilton Rating Scale for Anxiety). The improvement in Y-BOCS score seen in this study was comparable to the improvement reported in a meta-analysis of DBS studies (45.1% reduction in Y-BOCS score among 116 patients) (70). In another recent study, 10 patients (5 with refractory OCD, 5 with MDD) underwent MRgFUS anterior capsulotomy (71). In these patients, reduced symptoms of OCD (measured with Y-BOCS scores) and MDD (measured with Hamilton Rating Scale for Depression scores) were highly correlated with self-reported frontal function measured with the Frontal Systems Behavior Scale (72), thus indicating successful disruption of pathological function within the frontal-striatal networks by both up-regulating frontal-executive function and down-regulating OCD symptoms. However, this study reported no cognitive impairment after treatment, and therefore could not provide information regarding the upper limit of safety for lesion size. The results from these studies warrant large-scale and sham-controlled clinical trials to broaden our understanding of MRgFUS for the treatment of OCD.

### Epilepsy

Several clinical trials are ongoing to assess the use of FUS for the treatment of epilepsy, with some using low-intensity focused ultrasound (LIFU) to induce a neuromodulatory effect on the area with the highest epileptogenic activity within the temporal lobe and others using HIFU for thermoablation of a hypothesized epileptogenic focus using MRgFUS. Previous studies regarding FUS neuromodulation have led to the establishment of safe sonication parameters for reversible mechanical disruption of the neural circuit (73–75), and these induced neuromodulatory effects could be stimulatory and inhibitory depending on the targeted brain regions and pulsing schemes. In one clinical trial (NCT03657056, https://clinicaltrials.gov/ct2/show/NCT03657056), the BX Pulsar 1002 is being used to precisely target the epileptic focus in patients who are scheduled for temporal lobe resection. The feasibility and safety of the treatment with both excitatory and inhibitory LIFU parameters will be examined by assessing BOLD functional MRI signal changes throughout the LIFU procedure. Investigators in another clinical trial (NCT03868293, https://clinicaltrials.gov/ct2/show/NCT03868293) are also using the LIFU neuromodulatory effect to treat drug-resistant temporal lobe epilepsy. In this study, patients are undergoing a total of 8 LIFU treatment sessions within 1 month, with researchers assessing treatment efficacy by comparing the number of seizure episodes during and after treatment with the number of episodes in the pretreatment period. Investigators also hope to identify the electrophysiological changes in the epileptic tissue after LIFU neuromodulation and expect to reduce the frequency and/or attenuate the amplitude of epileptiform discharges recorded in electroencephalography data. The unique bimodal modulatory effect of LIFU intervention on the neuronal circuits that may initiate seizure activity may provide an important mapping strategy to identify the seizure focus when combined with electrophysiology or brain imaging readout. In another clinical trial assessing LIFU (NCT 02151175, https://clinicaltrials.gov/ct2/show/NCT02151175), investigators are again using the BX Pulsar 1002 device to study the excitatory and inhibitory effects of stimulation on patients with nondominant mesial temporal lobe epilepsy. The primary endpoint in this study is the safety of the device, which will be assessed by identifying any histological tissue changes. Secondary outcomes include changes in seizure frequency, neurological status, neuropsychological profile, and psychological profile. An initial publication from this group reported that among 8 patients who underwent LIFU sonication of the temporal lobe followed by resection of the affected side, no abnormal histological or neuropsychological changes were observed (76).

The results of another clinical study regarding the use of LIFU in the seizure onset zone were recently published (77). In this study, seizure focus was determined once patients had experienced at least 3 confirmed seizures after stereo-electroencephalography (SEEG) implantation. Patients then underwent LIFU using burst tone and nonthermal parameters with a 10-min exposure time. Sonication occurred while the SEEG electrodes were still implanted; they were removed 3 days later. Of the six patients who underwent treatment, three had no change in seizure frequency, two had a decrease in seizure frequency, and one had an increase in seizure frequency. More cases will need to be evaluated to determine the efficacy of LIFU as a treatment for patients with epilepsy. However, the use of LIFU should not be limited to treatment alone; variations in seizure activity can also aid in diagnosis and confirm the epileptogenic focus.

HIFU is also being investigated for the treatment of epilepsy. Patients with focal epilepsy are currently being recruited for a multicenter clinical trial (NCT02804230, https://clinicaltrials.gov/ct2/show/NCT02804230) that aims to evaluate the feasibility, safety, and initial efficacy of MRgFUS ablation of epileptic foci (defined in this study as temporal sclerosis, dysplasias, and heterotopias). Two other trials (NCT03417297, https://clinicaltrials.gov/ct2/show/NCT03417297 and NCT05032105, https://clinicaltrials.gov/ct2/show/NCT05032105) are assessing the feasibility and safety of using HIFU to ablate the anterior nucleus of the thalamus. One of the trials is focused on determining whether this ablation will help to prevent secondary generalization. The other is assessing the effect of ablation on focal seizure-related anxiety, using functional MRI to evaluate the reactivity of the amygdala to threat.

One of the challenges involved in treating epileptogenic lesions with HIFU is the size limitation of the ablation. Usually <1 cm in any diameter, the convergence of ultrasound waves in HIFU cannot completely ablate a large epileptogenic lesion in a single session. For instance, Yamaguchi et al. (78) described the use of HIFU to ablate a hypothalamic hamartoma in a 26-year-old man with medically refractory epilepsy and gelastic seizures. The hamartoma was too large for ablation, and so the case report details the authors' approach to achieve disconnection. First, electroencephalography was used to identify the location of the patient's seizure activity (right frontal lobe). Diffusion tensor tractography identified connectivity between the hypothalamus and right frontal lobe in the right posterior portion of the hamartoma. This boundary area was subsequently ablated with HIFU, creating a lesion with dimensions of 4.73 mm by 6.46 mm by SI (superior-inferior) 7.73 mm. The patient had no seizures after the ablation and remained seizure free over 1 year of follow-up. This case demonstrates the limitation of HIFU ablation size in epilepsy. However, with the integration of structural connectivity imaging, a disconnective approach could be the optimal treatment strategy.

Another challenge is that the most common type of epilepsy, mesial temporal lobe epilepsy, typically requires ablation of the anterior hippocampus and/or amygdala. Due to the incident angles of the ultrasound beam and the skull at this location, it is very difficult to achieve a high enough treatment efficiency to cause thermal ablation. One group in Japan published the first case report in thermal lesioning at the hippocampus for treating mesial temporal lobe epilepsy (12). Even with the maximum energy and high SDR (0.56), the temperature did not exceed ablative level, and postoperative MRI did not indicate any viable lesion. The patient remained seizure free after 12 months, and the authors theorized that there was a potential neuromodulatory effect due to the subablative temperature.

### Neuropathic Pain

The International Association for the Study of Pain defines the chronic pain indication as “persistent or recurrent pain lasting longer than 3 months” (79), at which point the pain network will no longer serve as a protective and healing mechanism but will be a pathological condition. Acute pain can occur in any part of the body, but as the pain evolves into a chronic state, pain information from the periphery to the thalamus will drive changes in higher-order brain areas, including reward, motivation, and cognition (80). Altering these widespread brain networks will change the biochemistry of pain transduction and affect the patient's cognitive and emotional experience in pain perception. Unfortunately, the current status of pain management using pharmacotherapy alone is limited to achieving satisfactory pain relief, and the conventional noninvasive brain stimulation modality is still controversial and lacks good scientific data to prove the effectiveness of the treatment (80).

MRgFUS has therapeutic potential for pain management using ablative therapy. In one sham-controlled randomized clinical study (NCT05122403), patients with medication-refractory neuropathic pain are undergoing MRgFUS central lateral thalamotomy followed by a double-blinded assessment regarding treatment effects and adverse events 3 months after treatment. Another ongoing clinical trial (NCT03309813) is targeting bilateral thalamic nuclei with MRgFUS to reduce pain and increase quality of life in patients with chronic trigeminal neuropathic pain. The goal of this randomized, sham-controlled, crossover study is to evaluate the safety and feasibility of treating chronic pain using the MRgFUS lesioning procedure.

## Fus-Mediated BBB Opening

### Brain Tumor

Among brain tumors, glioblastoma multiforme (GBM) is the most aggressive and is known to respond poorly to conventional chemotherapies. Contributing to this difficulty is the presence of the BBB, the tight junctions of which impair access of the macromolecular agents into the cellular environment. Additionally, the integrity of the BBB is highly heterogeneous due to the tumor microenvironment (81). Researchers initially found that HIFU could modify the BBB through the use of high intensities similar to those used to create lesions in normal tissue; however, this also led to an increased frequency of hemorrhage and edema (82, 83). Later research demonstrated that a reduction in the acoustic intensity could still achieve BBB opening with a lower incidence of unwanted side effects (84). In 2001, Hynynen et al. (85) found that with the intravenous injection of microbubbles, low-power FUS (ie, within the range of diagnostic ultrasound level, <1.5–2 MPa) could induce transient, reproducible, and localized BBB opening in rabbits without producing any associated neuronal damage. This study opened the door to improved chemotherapy delivery for patients with malignant brain tumors, with this technique allowing successful delivery of chemotherapeutic agents and thereby improving on the low (5%) efficacy of conventional systemic administration (86, 87). The FDA-approved use of concurrent microbubbles as contrast agents in diagnostic imaging thus permits safe doses of FUS intensity to disrupt the BBB (88, 89).

The first-in-human BBB opening was achieved using a transducer surgically implanted into the epidural space superficial to the tumor in patients with recurrent GBM (Table 1) (90). Patients received monthly FUS treatments coupled with intravenous injection of microbubbles for BBB opening. The pressure amplitude began at 0.5 MPa and increased to 1.1 MPa through 5 different doses (0.5, 0.65, 0.8, 0.95, 1.1 MPa), and BBB disruption was found to occur at pressure amplitudes >0.8 MPa. Disruption of the BBB was quantified with gadolinium-enhanced T1-weighted MR images; this technique was chosen based on data from a previous BBB opening study in nonhuman primates (91). Carboplatin, a common chemotherapeutic agent, was used in this study to control the recurrent GBM. This study was limited by the need to surgically implant the ultrasound transducer. Additionally, the transducer was unfocused and unable to electronically steer the beam after surgical implantation.

**Table 1:** FUS and microbubble parameters for BBB opening.

**Condition**	**Study**	**FUS system**	**FUS frequency**	**FUS intensity**	**MB dose**	**Sonication duration**	**Follow-up**	**Outcome**
Recurrent GBM	Carpentier et al. (90)	SonoCloud	1.05 MHz	0.5–1.1 MPa (>0.8 MPa opened the BBB)	Sonovue (0.1 mL/kg, with a maximum dose of up to 8.7 mL)	2.38% duty cycle for 150 s	6 mo	BBB of each patient was disrupted monthly using pulsed ultrasound in combination with systemically injected microbubbles
	Mainprize et al. (92)	The ExAblate Neuro	0.22 MHz	4–15 W	Definity (4 μL/kg); did not exceed 20 μL/kg	0.74% duty cycle for 50 s	3 mo	Reversible and safe opening of the BBB
	Park et al. (93)	The ExAblate Neuro	0.22 MHz	Maximum power did not exceed 40 W	Definity (4 μL/kg); did not exceed 20 μL/kg	5% duty cycle for 90 s	1 mo	Repetitive MRgFUS at the same target with standard chemotherapy
	Chen et al. (94)	NaviFUS	0.5	0.5–1.1 MPa (equates to 4, 8, 12 W)	Sonovue (0.1 mL/kg)	10 ms burst duration for 120 s. PRF of 9 Hz	36 ± 3 d	Feasibility and the tolerated dose of transient opening of the BBB
AD	Lipsman et al. (95)	The ExAblate Neuro	0.22 MHz	3–10 W	Definity (4 μL/kg); did not exceed 20 μL/kg	0.74% duty cycle for 50 s	2 mo	Reversible, safe, and repeated opening of the BBB

**Figure 11:**
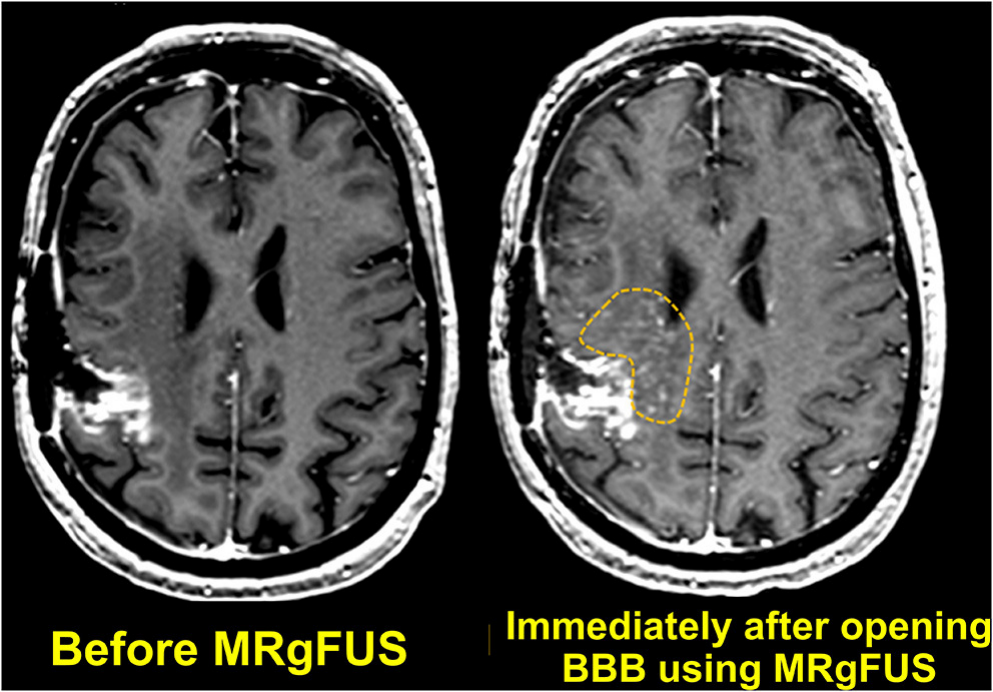
Axial T1-weighted MR images obtained before and immediately after MRgFUS was used to open the BBB. Localized extravasation (inside the dotted yellow circle) can be seen in the predefined targeted brain tissue.

A few years later, researchers performed a first-in-human trial of noninvasive MRgFUS BBB opening in patients with malignant glioma, using concurrent systemic administration of temozolomide chemotherapy (Table 1) (92). T1-weighted MR images demonstrated a 15% to 50% increase in signal enhancement, indicating transient BBB opening in the target tissue (Figure 11). Approximately 24 h after FUS and chemotherapeutic administration, the patients underwent craniotomy and tumor resection. Sonicated and unsonicated peritumor tissue samples were collected and the tissue chemotherapy concentrations were measured. Note that during the trial, the chemotherapy agent was switched from liposomal doxorubicin to temozolomide, and limited resectable tumor volume in three of five patients prevented statistical analysis of the tumor samples. Nevertheless, the researchers observed a chemotherapy concentration that was 7.7 times higher in the sonicated peritumor tissue than in the unsonicated peritumor tissue in one patient.

Another group of researchers subsequently tried to enhance the treatment effect by creating multiple BBB openings with MRgFUS (Table 1) (93). In this study, 6 patients who underwent a gross total resection of malignant glioma received 6 cycles of temozolomide with associated FUS BBB opening performed at the beginning of each 4-week cycle. Patients underwent follow-up MRI 1 year after the first chemotherapy cycle (6 months after the last chemotherapy cycle), and there was no evidence of any FUS-related adverse effects.

In 2021, another study demonstrated the feasibility and safety of using NaviFUS, a frameless novel device that integrates neuronavigation and an FUS system, in patients with GBM (94). Six patients were assigned to one of three different ultrasound doses in the mechanical index (0.48, 0.58, or 0.68) to temporarily open the BBB. The lowest dose used (0.48) was previously identified as the threshold of BBB opening in nonclinical studies (96); the maximum dose of 0.68 was chosen based on Good Laboratory Practice safety tests (97). Dynamic contrast-enhanced MRI was performed immediately after and 24 h after the BBB opening procedure and demonstrated the efficacy of NaviFUS BBB opening. T2-weighted images were obtained to evaluate any hemorrhages associated with BBB opening. All patients were scheduled for tumor resection surgery within 2 weeks after the FUS BBB opening, and clinical visits for follow-up were performed routinely until the third week after BBB opening to assess physical and neurological functions.

### AD and ALS

AD represents an enormous societal and healthcare burden as the population ages. Still, the development of new pharmacotherapeutics provides diminishing returns, as these drugs are restricted from entering the brain by the BBB. FUS temporarily loosens the BBB tight junction, allowing the delivery of therapeutic agents to the sonicated brain area. However, even without these therapeutics, studies have reported that BBB opening alone triggers a significant reduction of Aβ deposition through microglia activation (98, 99). Therefore, researchers have assessed the use of potential therapeutics such as a GSK-3 inhibitor and RN2N as an additive strategy that can further increase the therapeutic benefit of BBB opening (100). These preclinical studies using transgenic mouse models of AD have demonstrated improvements in Aβ plaque clearance and up-regulation of cognitive function in AD pathology after opening of the BBB. Based on these findings, researchers assessed the use of FUS in 4 patients with AD, and clinical and radiographic evaluations in these patients demonstrated reversible, repeatable, and safe noninvasive opening of the BBB with FUS (Table 1) (95). The researchers in this study targeted the superior frontal gyrus white matter of the dorsolateral prefrontal cortex to reduce the risk of adverse events. Note that a [18F]-florbetaben positron emission tomography scan performed 1 week after both the first and second sonication could not demonstrate a clear effect of BBB opening on Aβ clearance. Additionally, the small sample size limited the conclusions that could be drawn regarding the safety and efficacy of this treatment, as well as whether FUS BBB opening affected the clinical and pathological symptoms of AD.

Another research group developed a novel strategy in which FUS was delivered to deep brain regions without tissue ablation or BBB opening (101). The researchers used single ultra-short pulses (3 μs) instead of conventional pulses (100 ms) (75, 102) at 5-Hz pulse repetition frequency, and used 6,000 pulse numbers per session. This approach was found to be safe and effective in a preclinical study using an energy level of 0.3 mJ mm^−2^; the energy level was decreased to 0.2 mJ mm^−2^ for clinical purposes. In this study, 35 patients with mild AD treated at 2 separate clinics underwent FUS. In patients from clinic 1, researchers targeted the AD brain network, which included the dorsolateral prefrontal cortex, inferior frontal cortex, and language areas extending to Broca's and Wernicke's areas. In patients from clinic 2, researchers performed global cortical stimulation by distributing the total sonication energy over all brain areas by moving the headpiece probe over the scalp in a circular trajectory. Neuropsychological changes after therapy were evaluated with Consortium to Establish a Registry for Alzheimer's disease (CERAD) scores. Study patients demonstrated significant improvements in CERAD corrected total scores and logistic regression scores after treatment, and these improvements remained consistent over 3 months. Principal component analysis was also performed to assess CERAD-derived cognitive measurements of learning and memory, verbal skills, and visuospatial processing. Patients from clinic 1 demonstrated improvements in learning/memory and verbal skills lasting up to 3 months, and showed a decline in visuospatial processing. However, patients from clinic 2 demonstrated no significant change in visuospatial processing. This absence of stimulation effect could be due to the lack of stimulation of the occipito-parietal region in patients from clinic 2.

FUS also holds potential for the treatment of ALS. ALS is a devastating and incurable neurological illness, and medical advances have been incremental. As with brain tumors, the BBB is a pharmacologic barrier to potentially effective treatments for ALS. To this end, researchers assessed the use of MRgFUS to open the BBB in patients with ALS and demonstrated successful results (103). In this research, the brain region targeted for BBB opening was the eloquent primary motor cortex, and the process was found to be safe, feasible, and reversible. For patients with ALS, BBB opening is used to introduce agents such as nonviral vectors that transport therapeutic genetic elements into neurons; it is therefore essential that these agents are not damaged as they travel through the BBB (104).

## LIFU for Psychiatric Disorders and Impaired Consciousness

The introduction of LIFU, an incisionless brain stimulation modality that influences brain activity through subthermal temperature increases, presented a new opportunity to reversibly explore psychiatric disorders related to perception, emotion, and cognition along with altered states of consciousness. One of the first reports of the effects of transcranial ultrasound was published in 2013 and involved a double-blind crossover study of patients with chronic back pain (105). In this study, patients underwent either FUS or a sham session on a LOGIQe ultrasound imaging system with an 8-MHz probe placed over the frontal-temporal cortex contralateral to the side of maximal pain for 15 s. Forty minutes later, patients were switched to the opposite treatment arm (FUS or sham) for the second session. The parameter selection produced a mechanical index of 0.7 and a thermal index of 0.5, well below the FDA guidelines of 1.9 for mechanical index and 6.0 for thermal index. Patients in this study reported significant improvements in mood (measured with the Global Affect score derived from the Visual Analog Mood Scale) both 10 min and 40 min after FUS compared to the sham session.

Small animal studies using LIFU have suggested that targeted ultrasound could be used to restore consciousness after injury, although translating these results to humans is challenging because of the vast differences in scale between the awake state of humans and animals. The thalamus is often the target of choice in this research given its perceived role in the coordination of awake and sleep states. For example, Yoo et al. (106) demonstrated that performing thalamic LIFU led to a faster recovery time from ketamine/xylazine anesthesia in rats. In 2016, a case study was published reporting improvements in Glasgow Coma Scale and Coma Recovery Scale-Revised scores in a patient suffering from a posttraumatic disorder of consciousness (107). In this case, 10 pulsed sonications using a frequency of 650 kHz, an intensity of 720 mW/cm^2^ (I_SPTA_), and a pulse duration of 0.5 ms were applied to the thalamus. Each pulse train continued for 30 s with a subsequent 30-s interval. Five days after the sonication treatment, the patient attempted to walk and showed new motor responses and vocalization. The sonication parameters used in this study to stimulate the human thalamus were adapted from a previous rodent study where thalamic stimulation reduced the time under anesthesia (106). Except for the acoustic intensity being increased from 300 mW/cm^2^ to 720 mW/cm^2^ when translated to the human, fundamental frequency (650 kHz), duty cycle (5%), and pulse-repetition frequency (100 Hz) stayed the same. Based on the study in Plaskin et al. (108), a simulation model called neuronal intramembrane cavitation excitation (NICE) showed that thalamic reticular neurons display cell-type-specific inhibitory response to FUS parameters comprising 5% duty cycle and 100 Hz pulse-repetition frequency (PRF) driven by the particular membrane property of mechanosensitive T-type calcium channels. Those particular thalamic neurons are hypersensitive to a discontinuous pulsed mode of ultrasound stimuli compared to continuous mode as the T-type voltage-gated calcium channels show strong response during the hyperpolarization phase, and the depolarization phase results in increased calcium currents during the ultrasound off-period. Furthermore, the slow deactivation of the T-type calcium channel after the hyperpolarization allows charge accumulation during the ultrasound-off period and makes them more sensitive for re-excitation for repeated short-bursts of ultrasound pulses. Therefore, the authors believe that the neuromodulation effect on thalamic nuclei could modulate thalamocortical communication. In contrast, lesioning procedure uses continuous (100 % duty cycle) FUS parameters with the acoustic intensity at the thermoablation level. The researchers in this case sonicated the thalamus to modulate the cortico-striato-pallido-thalamo-cortical circuit; this decision was based on previous research in which the thalamus was targeted via pharmacological intervention (109), DBS (110), or transcranial direct current stimulation (111). The neuromodulatory effect, targeting, and safety of applying FUS to deep subcortical human brain was further assessed by Legon et al. (102). In this study, the authors found that sonicating the human thalamus reduced the amplitude of somatosensory evoked potentials and induced measurable behavior changes.

The use of LIFU has also been assessed for the treatment of mood disorders, in part because of the high prevalence of these disorders in the general population and inconsistent benefits with pharmacological treatment. Recently, researchers investigated whether FUS of the right inferior frontal gyrus (rIFG), a brain area associated with emotional regulation (112), could affect the mood of healthy participants in a randomized, placebo-controlled, double-blind study (113). Analysis of the participants' functional MRI results and self-reported moods demonstrated that FUS applied to the rIFG significantly enhanced mood for up to 30 min and significantly reduced specific brain connectivity between the rIFG and subgenual cortex for 20 min after sonication. These findings support previous research suggesting that interconnectivity between diverse brain regions is involved in the regulation of emotional and cognitive function (114–116). Previous research has also found that hyperactivity in the subgenual cortex is correlated with negative emotional states and might contribute to mood disorders such as depression (117). This study of FUS applied to the rIFG also demonstrated a decrease in default mode network connectivity (113). It is hypothesized that overexcited default mode network connectivity is associated with a lack of self-referential processing and the rumination that is frequently observed in patients with depression (118, 119). Therefore, depressive symptoms may be improved by down-regulating the activity of the rIFG with FUS. This research group subsequently reported research in which the same brain location was targeted in patients diagnosed with mild to moderate depression (120). In this study, they lowered the intensity of the ultrasound treatment from 130 to 71 mW/cm^2^ (I_SPTA_) delivered over 5 days. Patients who were treated with FUS self-reported a decrease in worry and an increase in happiness; however, the mood change was not consistent throughout the treatment period. The authors stated that the lower ultrasound intensity might be the reason for the inconsistent treatment effect and suggested that future studies should address parameter optimization to balance safety and efficacy. Nevertheless, this study shows that FUS may be a potential therapeutic intervention for depression, as only slight increases in worry and anxiety increase depression severity and the likelihood of refractory depression (121, 122).

## Future Outlook

Over the past 5 years, we have witnessed a global surge of publications regarding the clinical use of FUS across diverse neurological disorders, and this exponential growth of interest in the therapeutic potential of this modality has laid the foundation to optimize current technologies for human research. For instance, MR thermometry is crucial in MRgFUS lesioning procedure, as it provides real-time feedback of both anatomical location of the sonicated tissue and imposed thermal dose at the focus, which allow us to estimate the target accuracy and the degree of tissue damage for achieving thermal necrosis with precise spatial resolution. On the other hand, FUS can deliver exceptionally safe and stable opening of the BBB using passive cavitation detection, which is incorporated in MRgFUS system to real-time monitor the bubble activity provided by passive cavitation maps. Furthermore, functional connectivity is another modality of MRI often for examining the effect of FUS-mediated neuromodulation on network levels.

Besides the different types of MR tools coupled with the FUS system, each thermoablation therapy and BBB opening uses different FUS systems of MRgFUS. Although both FUS systems have hemispherical phased-array transducers consisting of thousands of elements, each is operated at different fundamental frequencies. 650 kHz FUS and 220 kHz systems are optimized for thermoablation and BBB opening, respectively. Using a high frequency of 650 kHz with a short wavelength and high intensity in a continuous waveform is useful when strategizing heat accumulation for irreversible tissue ablation. Using 650 kHz with 2.3 mm wavelength (assuming a speed of sound as 1,500 m/s in the brain) in a phased-array system enables tight focus and sharp demarcation within Vim, which is approximately 6–10 mm. On the other hand, using a low frequency of 220 kHz with a larger wavelength (6.8 mm) and burst-type of low-intensity in the pulsed waveform is beneficial when transiently opening the BBB within a large and complex target volume.

Researchers are also increasingly interested in the development of treatment methods that use the mechanical bioeffects of FUS, as there is a lower risk of thermal damage. Research in animal models has shown that fine-tuning of the pulse repetition frequency and the pulse duration with extremely high energy of ultrasound can create microbubble clouds to fractionate soft tissue (123–125), a method know as histotripsy. However, the mechanism of mechanical ablation is still poorly understood compared to the mechanism of thermoablation, and additional examinations of safety are still needed. Nevertheless, mechanical ablation is currently being explored for the treatment of stroke/intracranial hemorrhage (126–129). As with past research in this field, future studies will again require the expertise of the medical physics, imaging, engineering, and neuroscience communities.

## Conclusion

In conclusion, since the early attempts to use FUS-induced heating as a direct surgical approach in neurosurgery, acoustic energy in therapeutic applications has been widely attractive in diverse central nervous system diseases. Subsequently, therapeutic ultrasound has been under active investigation in preclinical and clinical studies for the last few decades. These research efforts marked the first successful culmination as experts in medical physics and engineers demonstrated the clinical feasibility of harnessing ultrasound energy by designing a transducer that can produce a focused beam of concentrated energy into the size of a grain of rice. This tight focus of concentrated acoustic energy allows FUS-induced heating to create a lesion at the particular region of the brain circuits responsible for pathological indications to normalize its function. Following the FDA approval of MRgFUS unilateral thalamotomy for ET in 2016 and MRgFUS unilateral pallidotomy for PD 5 years later, MRgFUS became a commercially available treatment option in the clinic for both ET and PD. Researchers and teams of clinicians—neuroradiologists, neurologists, and neurosurgeons—now envision extending FUS-mediated thermal ablation to treat a broader range of central nervous system diseases such as epilepsy, OCD, and neuropathic pain. FUS-mediated BBB opening combined with drug delivery is another promising modality requiring a team effort of broad interdisciplinary collaborations to fill the translation gap. Potentially, BBB opening technology could be developed into a localized therapeutic delivery and cellular delivery platform releasing chemotherapeutics, drug-encapsulated nanoparticles, stem cells, and immune cells for treating diverse neurological diseases.

Here we have reviewed the current clinical application of MRgFUS in treating brain disease in terms of thermoablation and BBB opening and discussed a growing number of clinical studies on FUS neuromodulation.

## Author Contributions

HB wrote the original draft of this review. SJ conceptualized and created all figures. SJ and SN substantively reviewed the final manuscript. All authors made contributions to sections, were involved in revising and editing the manuscript, and they all approved the final version of the manuscript.

## Conflict of Interest

EM was employed by InSightec, Inc. The remaining authors declare that the research was conducted in the absence of any commercial or financial relationships that could be construed as a potential conflict of interest.

## Publisher's Note

All claims expressed in this article are solely those of the authors and do not necessarily represent those of their affiliated organizations, or those of the publisher, the editors and the reviewers. Any product that may be evaluated in this article, or claim that may be made by its manufacturer, is not guaranteed or endorsed by the publisher.

## Abbreviations

AD: Alzheimer's disease
ALS: amyotrophic lateral sclerosis
BBB: blood-brain barrier
CERAD: Consortium to Establish a Registry for Alzheimer's Disease
CRST: Clinical Rating Scale for Tremor
CT: computed tomography
DBS: deep brain stimulation
ET: essential tremor
FDA: Food and Drug Administration
FUS: focused ultrasound
GBM: glioblastoma multiforme
HIFU: high-intensity focused ultrasound
HU: Hounsfield unit
LIFU: low-intensity focused ultrasound
MDD: major depressive disorder
MRgFUS: MR-guided FUS (MRgFUS)
MRI: magnetic resonance imaging
OCD: obsessive-compulsive disorder
PD: Parkinson's disease
SDR: skull density ratio
SEEG: stereo-electroencephalography
Vim: ventral intermediate nucleus
Y-BOCS: Yale-Brown Obsessive-Compulsive Scale.
